# *Alpinia*: the gold mine of future therapeutics

**DOI:** 10.1007/s13205-012-0089-x

**Published:** 2012-09-18

**Authors:** S. Ghosh, L. Rangan

**Affiliations:** Department of Biotechnology, Indian Institute of Technology Guwahati, Guwahati, 781039 Assam India

**Keywords:** *Alpinia*, Anticancer, Antioxidant, Bioactive compounds, Essential oil, Pharmacological

## Abstract

Since prehistoric era, plant-derived drugs were much preferred due to their less side effects than drugs of synthetic origin. Bioassay-guided selection of active fraction of a plant extract and further isolation and characterization of the pure bioactive compounds are in practice in both academic and industrial research. Zingiberaceae, a medicinally important, ornamental, monocotyledonous family has potential members in the tribe Alpinieae, among which the genus *Alpinia* is studied under this current review due to its wide range of biomedical applications. The members in the genus possess many bioactive compounds against harmful microbes to deadly diseases like cancer by regulating the different signalling pathway systems. Several compounds have been discovered and found to deliver diversified biological efficacy either in vitro or in vivo against a range of diseases. The chemical profiling of the genus and investigation of crude essential oils and individual bioactive compounds towards the therapeutic importance in various disciplines have been documented in the current review.

## Introduction

Plant-derived drug research has become more promising in recent years and also a better alternative for synthetic medicine and therapeutics in spite of many challenges (Vanwyk and Wink 2009). The bioactive natural compounds isolated from various parts of a plant are the key research thrust for a chemist, biologist, pharmacist, and medical professionals to tease and tap the potential of the so-called the ‘wonder’ molecules. In spite of great technological advancement in the field of applied science, medical treatments are still in its infancy when the treatment against the deadly diseases like cancer is considered. In many cases, it has been found that treatment of such diseases with the chemosynthetic drugs shows frequent side effects, toxicity, severe mental and physical abnormalities, not acceptable to the patient and to their families. Hence, the conservative mode of medical treatments and synthetic drugs available ‘off the shelf’ appears to be a serious concern.

Nearly, 21,000 plants have been listed by the World Health Organization (WHO), which are in use for diverse medicinal purposes around the world. Being the largest producer of medicinal herbs, India is known as the botanical garden of the world catering to the needs for herbal medicines (Seth and Sharma 2004). The WHO report revealed that around 80 % of world population depends on the traditional medicines, largely on plant-derived drugs towards their healthcare, among which 30 % of currently used therapeutics are from natural resources alone. Owing to the increasing cultural acceptability and significantly lower side effects, nearly 75–80 % of the whole population in the developing countries mostly prefers the herbal treatment for primary health care (Ghasi et al. 2000).

Ethnopharmacogological knowledge towards the scientific investigation of medicinally important plants augments the prospects of alternative medicine and therapeutic values. The ethnomedical practices of the tribal communities of North East India were critically studied and documented for the Zingiberaceae family towards their future pharmacological diagnostics (Tushar et al. 2010). This important family is distributed worldwide with about 50 genera and 1,300 diverse species mainly concentrating in South and Southeast Asia (Wu and Larson 2000). In India, about 22 genera and 178 species have been reported from North Eastern and peninsular region (Jain and Prakash 1995), whereas North East region alone harbours 19 genera and close to about 88 diverse species (Prakash and Mehrotra 1995). Latin and species description in many cases are in doubtful identity.

The largest genus of the family Zingiberaceae, *Alpinia*, was classified by Charles Plumier, the famous French botanist and named after Prospero Alpino, the well-known Italian botanist of sixteenth century. The genus, *Alpinia* belongs to the flowering plants group (angiosperms); as per the Angiosperm Phylogeny Group II (APG II) system, it comes under the umbrella of monocotyledonous plants (Angiosperm Phylogeny Group 2003), belonging to the order Zingiberales, subfamily Alpinioideae and tribe Alpinieae. The genus includes 230–250 species distributed throughout tropical and subtropical climates of Asia and the Pacific. DNA-based studies showed the genus as polyphyletic represented by six clades scattered across the tribe Alpinieae (Kress et al. 2005).

**Fig. 1:**
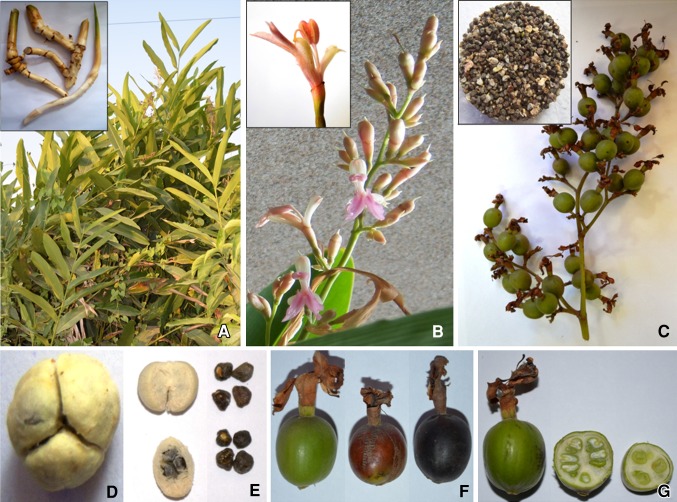
Different plant parts of *A. nigra* used to extract bioactive compounds. **a** Alternate phyllotaxy of plants, *inset* depicts the stolon type of rhizome; **b** racemose type of inflorescence, *inset* shows single flower, **c** developing fruit cluster, *inset* shows mature seeds, **d** pulpy dehusked fruit (trilocular), **e** locules and mature seeds, **f** different stages of fruit maturity and **g** longitudinal and cross-sectional view of the immature fruit

Majority of the members of the genus produces attractive inflorescence, possesses aromatic aerial and underground parts generally subjected to different fractionation process for the extraction of essential oils, aqueous extract and bioactive components. Various parts of this plant have significant potential to yield bioactive components towards the development of future therapeutics (Fig. 1). The essential oil extracted from different parts of the plant contains diverse natural compounds having multiple medicinal properties. Because of its multipurpose utility, the genus *Alpinia* demands much attention from the researchers towards the development of potential therapeutics against various diseases like cancer, diabetes, ulcer and many neural disorders. Several research and reviews shows the importance and medical application of potential bioactive compounds isolated from different species of the genus and further research is continuing to unveil the mechanism of action of the natural bioactive compounds in regulating the disease progression and cure. The current aim of this study is to highlight the exhaustive pharmacological information and promising therapeutic uses of the genus *Alpinia*.

## Isolation and characterization of natural bioactive compound (phyotochemistry)

The members of the genus *Alpinia* have complex chemical profiles and possess diverse flavonoids and are being considered as chemosystematic markers for the key identification and order classification (Pugialli et al. 1993). The flavonoids, in general, are known to be responsible for yellow pigmentation in plant tissues, and are potential source of antioxidants, many of which have anticancerous activities due to the presence of functional keto (C=O) or aldehyde (–CHO) groups (Williams et al. 2004). The aqueous and organic solvent extract harbours many bioactive compounds and their natural derivatives which differ from species to species and also plant parts used (rhizomes, stems, leaves, flowers, seeds and fruits) for isolation (Table 1).

**Table 1:** List of prospective pharmacologically important bioactive compounds isolated from different species of *Alpinia*

Species name	Plant parts used	Structure and name of the compounds	Bioactivities	References
*A. galanga*	Rhizome		Antifungal	Janssen and Scheffer (1985)
*A. mutica*	Rhizome		Anticancer	Malek et al. (2011)
*A. katsumadai*	Seeds		Anticancer	Hua et al. (2009)
*A. galanga*	Rhizome		Treatment against osteoarthritis	Phitak et al. (2009)
*A. oxyphylla*	Kernels		Neuroprotective activity	An et al. (2008)
*A. conchigera*	–		Inhibitor of NF-κB activation	Lee et al. (2006)
*A. officinarum*	Rhizome		Antiinflammatory	Yadav et al. (2003)
*A. blepharocalyx*	Seeds		Antiplatelet	
*A. speciosa*	Rhizome		Antioxidant	Masuda et al. (2000)
*A. zerumbet*	Leaves		HIV-1 integrase and neuraminidase inhibitors	Upadhyay et al. (2011)
*A. galanga*	Rhizome		Antileishmanial	Kaur et al. (2010)
*A. ligulata* and *A. nieuwenhuizii*	Rhizome		Antimicrobial	Yusoff et al. (2011)

Therefore, before exploitation of these natural compounds for diverse biological activities, isolation and characterization for each of them need to be done primarily by different spectral and analytical techniques. The isolation, chemical and molecular characterization of natural compounds has been done by standard bioanalytical techniques like preparative thin layer chromatography (pTLC), column chromatography (CC), high performance liquid chromatography (HPLC), ultraviolet (UV) spectral analysis, fourier transform infrared spectroscopy (FTIR), ^13^C and ^1^H nuclear magnetic resonance (NMR) and mass spectroscopy (MS). Till date, several bioactive compounds have been isolated and characterized from different species of the genus *Alpinia*. Some notable are enlisted in Table 1.

## Bio-pharmaceutical potential

**Fig. 2:**
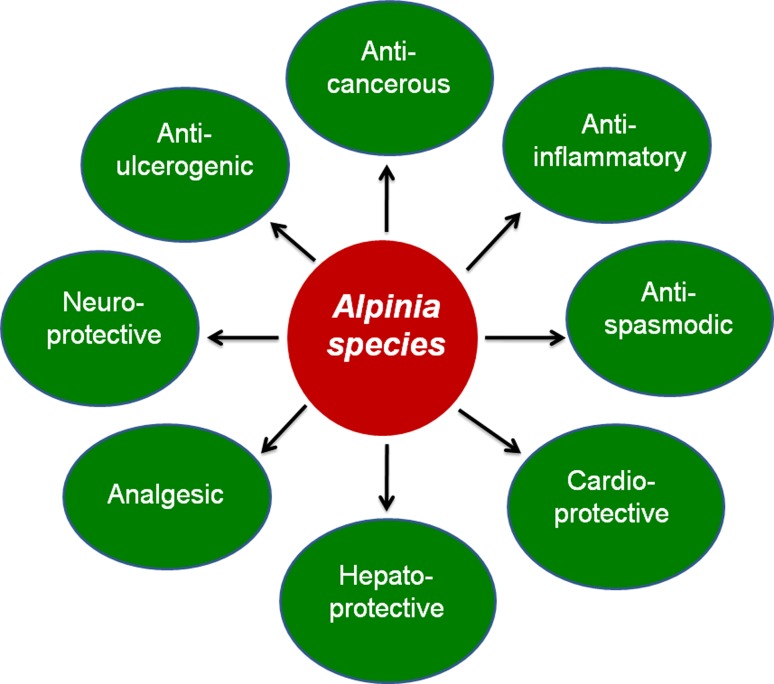
Diverse biomedical application of *Alpinia* species

Reviewing of the genus *Alpinia* showed its incredible biopharmaceutical potentials as evident from earlier published reports and is gaining the attention of researchers from different disciplines. The presence of the bioactive substances such as flavonoids, tannins and terpenes is the key for its therapeutic efficiency. The potential biomedical applications of diverse species of *Alpinia* are depicted in Fig. 2. Brief accounts of its biological efficacy towards the therapeutic uses are described below.

### Antimicrobial activity

A great depth of antimicrobial activities has been reported from *Alpinia* species having diverse chemical profile. Till date most of the work has been concentrated in *A. galanga* which contain more bioactive compounds compared to other species in the genera (Janssen and Scheffer 1985; Oonmetta-aree et al. 2006; Khattak et al. 2005; Weerakkody et al. 2011; Rao et al. 2010; Niyomkam et al. 2010). Essential oil extracted from fresh and dried rhizomes of *A. galanga* have potential antimicrobial activities against a range of bacteria, fungi, yeast and parasite. Ethanol extract from rhizome showed cytological modification to *Staphyllococcus aureus* cells by altering outer membrane integrity (Oonmetta-aree et al. 2006). However, the galangal extract, being hydrophobic in nature, could not inhibit the proliferation of gram-negative bacteria as the extract unable to penetrate the lipopolysaccharide monolayer of outer membrane of the cell wall. Terpinen-4-ol, a monoterpene, purified from the essential oil of fresh galangal rhizomes, showed antimicrobial activity against *Trichophyton mentagrophytes*. Similarly, acetoxychavicol acetate (ACA) isolated from dried rhizomes of *A. galanga*, is potentially active against several bacteria and many dermatophytes (Janssen and Scheffer 1985).

Besides the *A. galanga*, other species, viz. *A. oxyphylla*, *A. speciosa*, *A. zerumbet* and many others are gaining attention due to the presence of diverse polyphenolic compounds and their complex chemical profile. Various studies showed the antimicrobial potential of crude ethanolic extract, chloroform extract, hydrodistillation extract and a number of purified compounds against a wide spectrum of microorganism (Table 2). Moreover, recent findings showed antiviral potential of diarylheptanoid from *A. katsumadai* seeds. The extracts showed in vitro neuraminidase inhibitory activities against human influenza virus A/PR/8/34 of subtype H1N1 (Grienke et al. 2010). Further, different fractions of ethanolic extract were found promising against A/Chicken/Korea/MS96/96 (H9N2) influenza viruses operated by inhibiting viral hemagglutinin binding to the sialic acid receptors in the host cell (Kwon et al. 2010). The significant antimicrobial activities of different fractions and pure components of *Alpinia* species are catalogued in Table 2.

**Table 2:** List of antimicrobial, antiparasitic and insecticidal actions of bioactive fractions and pure compounds of *Alpinia* species

Species name	Parts used	Bioactive fractions/compounds	Bioactivity	References
*A. galanga*	Rhizome	Acetoxychavicol acetate	Antifungal	Janssen and Scheffer (1985)
*A. katsumadai*	Seeds	Ethanol extract and fractions	Antiviral	Kwon et al. (2010)
*A. conchigera*	Leaves, stem and rhizomes	Essential oil obtained from hydrodistillation	Antibacterial and antifungal	Ibrahima et al. (2009)
*A. galanga*	Rhizome	d,l-1-Acetoxychavicol acetate	Antimicrobial	Oonmetta-aree et al. (2006)
*A. galanga*	Rhizome	Ethanol extract	Antimicrobial	Khattak et al. (2005)
*A. galanga*	Rhizome	Chloroform extract	Antigiardial	Sawangjaroen et al. (2005)
*A. speciosa*	Leaves	Ethanol extract	Antimicrobial	Wang and Huang (2005)
*A. calcarata*	Rhizome	Hydrodistilled essential oil	Antifungal	Lakshmi et al. (2010)
*A. galanga*	Rhizome	Ethanolic extract	Antidermatophytic	Trakranrungsie et al. (2008)
*A. speciosa*	Leaves	5,6-Dehydrokawain derivatives	Antifungal	Tawata et al. (1996)
*A. ligulata* and *A. nieuwenhuizii*	Rhizome	Essential oil	Antibacterial and antifungal	Yusoff et al. (2011)
*A. pahangensis*	Leaves and rhizomes	Hydrodistilled essential oil	Antibacterial	Awang et al. (2011)
*A. galanga*	Rhizome	1′-Acetoxy-chavicol acetate	Antibacterial	Weerakkody et al. (2011)
*A. galanga*	Leaves and rhizomes	Methanol, acetone and diethyl ether extracts	Antibacterial	Rao et al. (2010)
*A. galanga*	Rhizome	Ethyl acetate extract (1′-acetoxychavicol acetate)	Protects acne	Niyomkam et al. (2010)
*A. galanga*	Rhizome	Chloroform extracts	Antifungal	Phongpaichit et al. (2005)
*A. galanga*	Rhizome	Ethanolic extract	Antifungal	Ficker et al. (2003)
*A. galanga*	Rhizome	Chloroform extract	Antiamoebic	Sawangjaroen et al. (2006)
*A. nigra*	Shoots	Crude aqueous extract	Flukicidal	Roy and Tandon (1999)
*A. galanga*	Rhizome	Methanol extract	Antimalarial	Abdulelah et al. (2010)
*A. nigra*	Shoots	Ethanolic extract	Anthelmintic	Roy and Swargiary (2009)
*A. galanga*	Rhizome	Hexane, chloroform and ethyl acetate extract	Antileishmanial	Kaur et al. (2010)
*A. galanga*	Rhizome	Hexane, dichloromethane, ethyl acetate and ethanol	Insecticidal	Sukhirun et al. (2010)
*A. oxyphylla*	Fruits	Methanol extract, yakuchinone A (1)	Insecticidal	Miyazawa et al. (2001)
*A. oxyphylla*	Fruits	Nootkatone	Insecticidal	Miyazawa et al. (2000)
*A. purpurata*	Flowers	Essential oils and aqueous extracts	Larvicidal and antibacterial	Santos et al. (2012)

### Antiparasitic and insecticidal activity

**Fig. 3:**
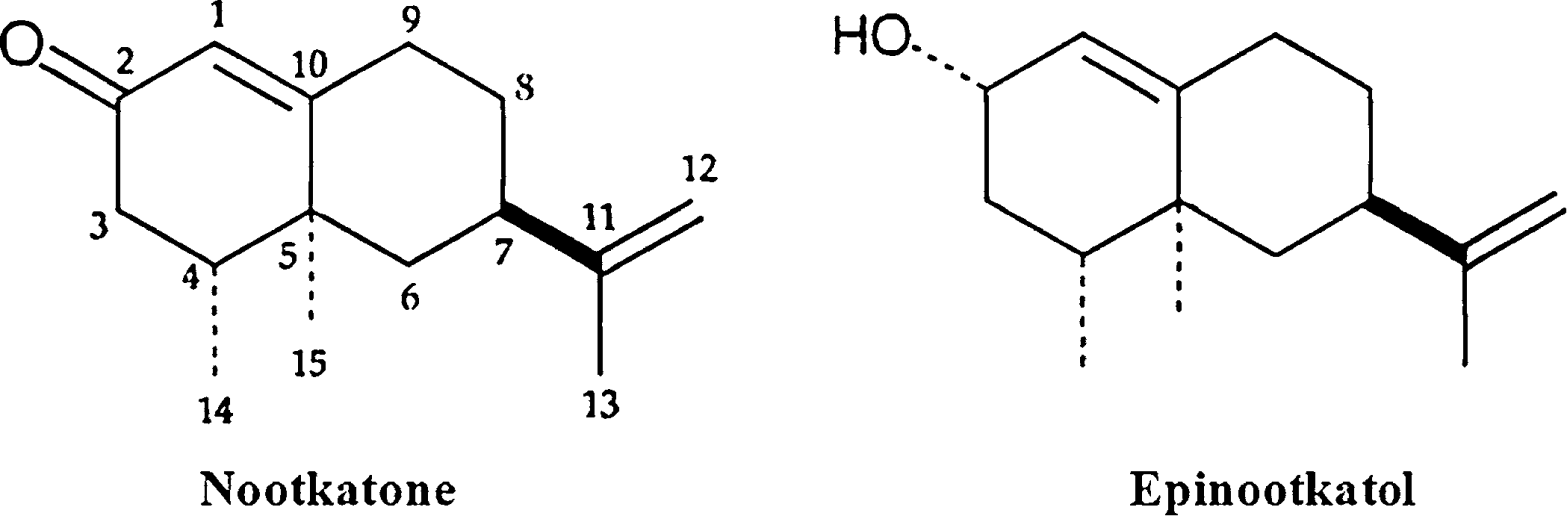
The structure of Nootkatone and Epinootkatol isolated from *A. oxyphylla* fruits, where they differ on their 2-position due to the presence of carbonyl (–C=O) and aldehyde (–CHO) group, respectively

Many parasites and insects pose severe threat to human and animal health. A number of medicinally important plants were tested towards their potential as an antiamoebic agent and it was found that the chloroform extracts from *A. galanga* to be highly effective with an added desired advantage of less side effects than traditional medicine, viz. metronidazole (Sawangjaroen et al. 2006). Miyazawa et al. (2000) reported that methanolic extract of *A. oxyphylla* was found to possess insecticidal activity against larvae of *Drosophila melanogaster* Meigen. From the crude extract, an insecticidal compound was separated by bioassay-guided fractionation and identified to be nootkatone by GC, GC–MS, and 1H and 13C NMR spectroscopy. Further, bioassay-guided studies for insecticidal activity, nootkatone showed a LC50 value of 11.5 μmol/mL of diet against larvae of *D. melanogaster* and a LD50 value of 96 μg/L against adults. Another compound, epinootkatol, however, showed moderate insecticidal activity in both assays, indicating that the carbonyl group at the 2-position in nootkatone was important for enhanced insecticidal activity (Fig. 3).

Recently, for the first time antileishmanial phenylpropanoids has been isolated using hexane, chloroform and ethyl acetate extracts of *A. galanga* rhizome (Kaur et al. 2010). Among several compounds purified, *p*-coumaryl diacetate, 1′-acetoxychavicol acetate, 1′-acetoxyeugenol acetate and *trans*-*p*-acetoxycinnamyl alcohol were found most promising in vitro against promastigotes of *L. donovani* with IC50 values of 39.3, 32.9, 18.9 and 79.9 μM, respectively. The genus, *Alpinia* harbour prospective compounds towards the antiparastic and insecticidal actions as enlisted in Table 2.

### Anticancerous activity

Many in vitro studies that have been done in diverse cancer cell lines and in vivo studies with animal models reflect clearly the potential of *Alpinia* species as anticancerous plant. For instance, a novel compound, Pinostrobin chalcone, has been isolated from *A. mutica* which displays notable cytotoxic potential to various human carcinoma cell lines (KB, MCF7 and Caski cells) with significant IC50 values (Malek et al. 2011). Antiangiogenic potential of *A. oxyphylla* fruits has been found in *n*-hexane and ethyl acetate fractions and tested against zebrafish model, human umbilical vein endothelial cells and tumour cell lines and have been hypothesized against cancer and inflammation diseases (He et al. 2010). Investigation of Nam et al. (2005) on the *n*-hexane and chloroform extract of *A. galanga* rhizome lead to the isolation of two compounds, viz. 1′-(*S*)-1′-acetoxychavicol acetate and *p*-coumaryl alcohol γ-*O*-methyl ether. Of the two compounds, former showed significant cytotoxic activity against human cancer cell lines like A549 (IC50 = 8.14 μg/mL), SNU638 (IC50 = 1.27 μg/mL), HT1080 (IC50 = 1.2 μg/mL), HL60 (IC50 = 2.39 μg/mL) and HCT116 (IC50 = 1.77 μg/mL). Whereas, the second compound revealed specific activity against SNU638 (IC50 = 1.62 μg/mL). In some other cancer cell lines cytotoxic activity has been screened with four different compounds isolated from *A. officinarum* and only 7-(3,4-dihydroxyphenyl)-1-(4-hydroxy-3-methoxyphenyl)-4-en-3-heptanone was found remarkable cytotoxic agent against HepG2, MCF-7 and SF-268 (An et al. 2008).

Lu et al. (2007) studied the effect of flavonoid constituents of *A. officinarum* on whitening effects based on melanin biosynthesis in B 16 mouse melanoma cells. The flavonoid mixture and galangin exhibited a broad absorption band at 270–290 nm related to the UV-B area supporting that galangin could be a whitening agent and a capable candidate for prevention of skin cancer. The summarized anticancerous activities of the crude extract and isolated principal compounds of the genus *Alpinia* are listed in Table 3.

**Table 3:** List of anticancerous, antiinflammatory and analgesic activities showed by bioactive fractions and major compounds from *Alpinia* species

Species name	Parts used	Bioactive fractions/compounds	Bioactivity	References
*A. galanga*	Rhizome	1′*S*-1′-Acetoxychavicol acetate and *p*-coumaryl alcohol γ-*O*-methyl ether	Anticancerous	Nam et al. (2005)
*A. officinarum*	Rhizome	7-(3,4-Dihydroxyphenyl)-1-(4-hydroxy-3-methoxyphenyl)-4-en-3-heptanone	Anticancerous	An et al. (2008)
*A. pricei*	Rhizome	Ethanolic extract	Apoptotic	Yang et al. (2008)
*A. oxyphylla*	Fruits	Oxyphyllone A and B	Anticancerous	Xu et al. (2009)
*A. conchigera*	Rhizome	1′*S*-1′-Acetoxychavicol acetate	Apoptotic	Awang et al. (2010)
*A. katsumadai*	Seeds	Rubraine, isorubraine and sumadain	Anticancerous	Hua et al. (2009)
*A. scabra*	Leaves and rhizome	Hexane and dicholoromethane extract	Anticancerous	Ibrahim et al. (2010)
*A. oxyphylla*	Fruits	Hexane and ethyl acetate fractions	Antiangiogenic	He et al. (2010)
*A. mutica*	Rhizome	Pinostrobin	Anticancerous	Malek et al. (2011)
*A. officinarum*	Rhizome	Galangin	Prevents skin cancer	Lu et al. (2007)
*A. blepharocalyx*	Seeds	Diarylheptanoids	Antiproliferative	Ali et al. (2001)
*A. calcarata*	Rhizome	Aqueous and ethanolic extract	Antinociceptive	Arambewela et al. (2004)
*A. officinarum*	Rhizome	Ethanolic extract	Antinociceptive, antiinflammatory, and antipsychiatric	Lee et al. (2009)
*A. officinarum*	Rhizome	Hydroxy-1,7-diphenyl-4-en-3-heptanone 6, 6-(2-hydroxy-phenyl)-4-methoxy-2-pyrone, 1,7-diphenyl-4-en-3-heptanone, 1,7-diphenyl-5-methoxy-3-heptanone and apigenin	Platelet-activating factor (PAF) antagonists	Fan et al. (2007)
*A. galangal*	Rhizome	Alcoholic and aqueous extracts	Antiinflammatory	Satish and Dhananjayan (2003)
*A. conchigera*	Rhizome	Cardamomin	Antiinflammatory	Lee et al. (2006)
*A. galanga*	Rhizome	7-(4′-Hydroxy-3′-methoxyphenyl)-1-phenylhept-4-en-3-one	Antiinflammatory	Yadav et al. (2003)
*A. galanga*	Rhizome	1′*S*-1′-acetoxychavicol acetate and 1′*S*-1′-acetoxyeugenol acetate	Antiallergic	Matsuda et al. (2003a, b)
*A. galanga*	Rhizome	Acetoxybenzhydrols	Antiallergic	Yasuharaa et al. (2009)
*A. pricei*	Rhizome	70 % Ethanolic extract	Antiinflammatory	Yu et al. (2009)
*A. pricei*	Rhizome	Flavokawain B	Antiinflammatory	Lin et al. (2009)

### Antiinflammatory and analgesic activity

Inflammation is a protective response by the organism to eliminate the injurious stimuli and to initiate the healing process. It’s a complex biological response of vascular tissues to detrimental stimuli such as pathogens, injured cells or external irritants (Ferrero-Miliani et al. 2007). Therefore, antiinflammatory drugs refer to the property of a substance that trims down inflammation. Antiinflammatory drugs reduce inflammation without affecting the central nervous system and make up about half of analgesics available in the market. Medication towards inflammation depends on steroids, non-steroidal antiinflammatory drugs (NSAID), immune selective antiinflammatory derivatives (ImSAIDs) and herbal drugs. However, inhibitions of natural hormones and liver dysfunction are the common side effects of steroidal drugs (Urhausen et al. 2003; Hartgens et al. 1996). Similarly, NSAID can cause gastric erosions, leading to stomach ulcers and in extreme cases can cause severe haemorrhage, resulting in death by myocardial infarction and stroke (Trelle et al. 2011). Therefore, ImSAIDs and herbal drugs are more acceptable to treat inflammation and remedying pain. There are several bioactive compounds that have been isolated from *Alpinia* species which shows antiinflammatory and analgesic actions.

Natural bioactive compounds and crude hydroalcoholic fractions isolated from the *Alpinia* species like *A. galanga*, *A. zerumbet*, *A. officinarum*, etc., showed potential activities as antiinflammatory and analgesic agent. Aqueous and hydroalcoholic extracts from leaves and rhizomes of above species possesses key factors responsible for antinociceptive (reducing sensitivity to painful stimuli) and antiallergic properties. Diarylheptanoids, a novel class of potent platelet-activating factor (PAF) antagonists from *A. officinarum* rhizome extract was recently identified (Fan et al. 2007), which also showed antirheumatic, antipsychiatric and analgesic activities with 80 % ethanolic extract (Lee et al. 2009). A brief account of the antiinflammatory, analgesic and other related activities of *Alpinia* are listed in Table 3.

### Neuroprotective activity

*A. galanga* has been exhaustively explored towards diverse biological activities in most of the cases among different *Alpinia* species. Recently, chloroform fraction of *A. galanga* has been found as antiamnesic probably due to the presence of 1′*S*-1′-acetoxyeuginol acetate as lead compound (Singh et al. 2011a). *A. oxyphylla* fruit was found to have the neuroprotective activities (Koo et al. 2004) and subsequently many other *Alpinia* species have been reported since (Table 4). Protocatechuic acid (PCA), a principal compound of the *A. oxyphylla*, protects against oxidative damage in vitro and reduces oxidative stress in vivo (Shi et al. 2006). It has been shown that PCA also reduces the hydrogen peroxide or sodium nitroprusside induced cell death in PC12 cells in dose-dependent manner (An et al. 2006) and this offers a valuable therapeutic strategy for the cure of oxidative stress-induced neurodegenerative disease like Parkinson’s disease. Other reports revealed that *A. katsumadai* seed extract protects neurons from ischaemic damage (Li et al. 2011a) and the treatment significantly decreased the activation of astrocytes and microglia in the hippocampal CA1 region (Li et al. 2011b). Similarly, methanolic extract of *A. officinarum* rhizome showed protection against oxidative damage in PC 12 cells (Chang et al. 2011).

**Table 4:** List of neuroprotective and antioxidant activities exhibited by various natural bioactive compounds and crude fractions of *Alpinia* species

Species name	Parts used	Bioactive fractions/compounds	Bioactivity	References
*A. oxyphylla*	Fruits	Ethanolic extract	Neuroprotective	Yu et al. (2003)
*A. oxyphylla*	Fruits	Protocatechuic acid	Neuroprotective	Shi et al. (2006)
*A. oxyphylla*	Kernel	Protocatechuic acid	Neuroprotective	An et al. (2006)
*A. officinarum*	Rhizome	Methanolic extract	Neuroprotective	Chang et al. (2011)
*A. katsumadai*	Seeds	70 % Ethanolic extract	Neuroprotective	Li et al. (2011a)
*A. katsumadai*	Seeds	Ethanolic extract	Neuroprotective	Li et al. (2011b)
*A. oxyphylla*	Fruits	80 % Ethanolic extract	Neuroprotective	Zhang et al. (2011b)
*A. oxyphylla*	Fruits	Water extract	Neuroprotective	Koo et al. (2004)
*A. oxyphylla*	Fruits	94 % Ethanolic extract	Neuroprotective	Yu et al. (2003)
*A. galanga*	Rhizome	*n*-Hexane, chloroform and ethyl acetate	Neuroprotective	Singh et al. (2011a)
*A. galanga*	Rhizome	Ethanolic extract	Neuroprotective	Singh et al. (2011b)
*A. zerumbet*	Leaves and rhizome	Dihydro-5,6-dehydrokawain and other ethyl acetate and hexane extract	Antioxidant	Elzaawely et al. (2007b)
*A. zerumbet*	Flowers and seeds	Ethyl acetate and hexane extract	Antioxidant	Elzaawely et al. (2007a)
*A. galanga* and *A. allughas*	Rhizome	Dichloromethane and methanol extract	Antioxidant	Vankar et al. (2006)
*A. speciosa*	Rhizome	Feruloyl esters with epicatechin	Antioxidant	Masuda et al. (2000)
*A. katsumadai*	Seeds	Epigallocatechine-3-gallate, resveratrol and total extract	Antioxidant	Lee et al. (2003)
*A. officinarum*	Rhizome	Methanolic extract	Antioxidant	Chang et al. (2011)
*A. calcarata*	Rhizome	Hydrodistilled n-pentane and ether extract	Antioxidant	Arambewela et al. (2010)
*A. oxyphylla*	Fruits	Protocatechuic acid	Antioxidant	Zhang et al. (2011a)
*A. galanga*	Rhizome	Ethanol extract	Antioxidant	Singh et al. (2011b)
*A. densespicata*	Stem and leaves	Ethanol extract	Nitric oxide inhibitory	Kuo et al. (2009)
*A. officinarum*	Rhizome	Hydro alcoholic extract	Antioxidant	Srividya et al. (2010)

### Antioxidant and other activities

Essential oil of *A. zerumbet* has strong potential as antipsychotic and antioxidant agent (de Araújo et al. 2011) which may have promising efficacy for the treatment of schizophrenia. On the other hand, *A. galanga* ethanol extract shows antiamnesiac effect in Amyloid β induced neurodegeneration (Singh et al. 2011b). Members of the *Alpinia* genus are found to have a remarkable antioxidant activity which in turn gives more biological efficacy towards the development of therapeutics. The antioxidant activities of the genus are enlisted in Table 4.

Besides above activities, the genus is also emerging as the prospective source for antiageing compound which is found to be PCA from *A. oxyphylla* (Zhang et al. 2011a). Aqueous acetone extract of *A. officinarum* rhizome showed inhibition to melanogenesis process (Matsuda et al. 2009), whereas acetone extract of *A. oxyphylla* fruits acts as a potent skin permeation enhancer (Fang et al. 2003). Recent studies revealed two bioactive compounds from *A. zerumbet* rhizome and leaves, viz. 5,6-dehydrokawain (DK) and dihydro-5,6-dehydrokawain (DDK). The compounds were found to be potent inhibitor of HIV-1 integrase and neuraminidase (Upadhyay et al. 2011) indicating that it could be used as potent drugs against those viral diseases.

## Future perspective and consideration

**Fig. 4:**
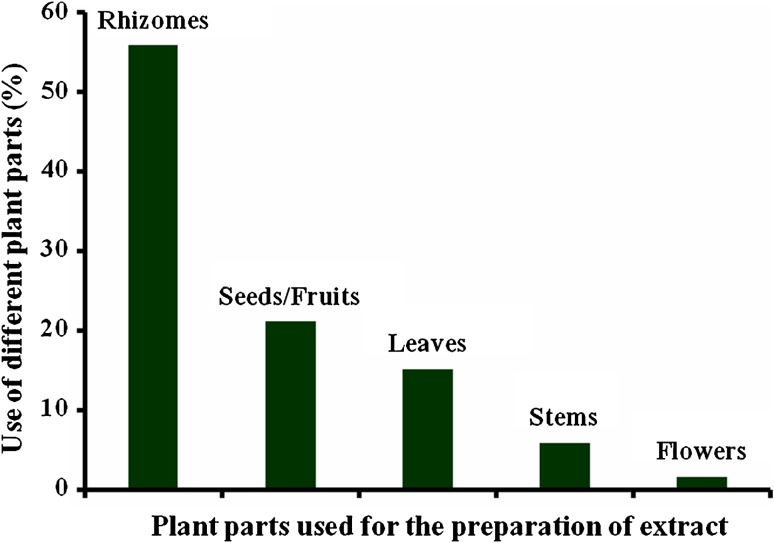
The graph represents the use of various plant parts in terms of percent use towards the versatile bioactivity studies under consideration in the current review

**Fig. 5:**
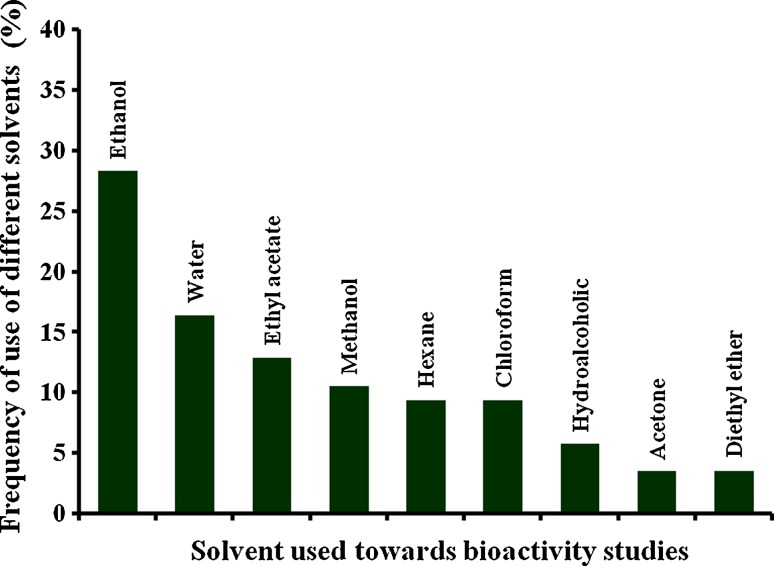
Different solvent system has been used for the extraction of crude oil and bioactive components. Each *bar* represents the percentage of uses of each solvent towards the extraction method related to the genus *Alpinia* documented under current study

**Fig. 6:**
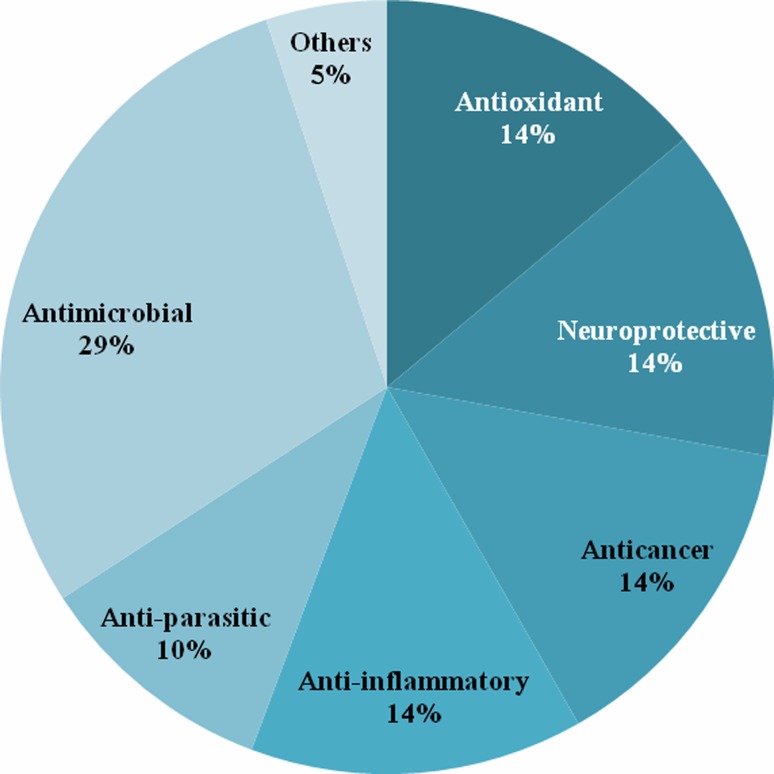
The potential application and research carried out in various field of biomedical sciences related to the genus *Alpinia* and expressed in terms of percentage biological activities considered under the current study

In the current study, it has been observed that various plant parts of different *Alpinia* species are used to get the bioactive compounds and different fractions show remarkable biological efficacy against various biomedical challenges. Detailed examination of the gathered data in *Alpinia* shows that rhizome is the main plant part used for pharmacological investigation, whereas other vegetative and reproductive parts were used moderately (Fig. 4). Most of the cases it has been observed that rhizomes harbour most of the essential oil components and showed potential biological activities at different scale. It has also been observed that various solvent systems were used in the bioactivity studies and isolation of bioactive compounds from the plant parts which acts as a key factor in terms of yield, number of compounds, type of compounds, etc. In the current study, it has been clearly observed that ethanol fraction has been the most preferred solvent system which has been used either in the initial crude oil extraction or in the further fractionation process (Fig. 5). The aqueous solvent was found to be the second best choice for the study as it also can extract copious amount of essential oil from different plant parts, but it varies from species to species. The bioactive compounds or crude fractions of essential oils from various species of *Alpinia* were found to be promising against various biomedical challenges like antimicrobial, anticancerous, antileshmania and many more. Also, in the current study, it has been observed that various species of *Alpinia* has ample potential to overpower biomedical threats including the most diverse microbes in the mother earth. Moreover, the genus *Alpinia* harbours versatile components towards its diverse biological efficacy (Fig. 6). Much more understanding and further exploration will be needed towards the other unexplored species of the genus, viz*. A. nigra*, *A. katsumadai*, *A. pahangensis*, *A. nieuwenhuizii* and many more to circumvent the future biomedical challenges.

## Conclusion

Detailed account of the diverse utility of the genus *Alpinia* can be addressed, starting with the ethnomedicinal information culminating with exhaustive scientific exploration. Towards the pharmacological investigation and future diagnostics, drug designing and modulating different trans-regulating pathways will be useful to fight against the deadly diseases prevalent in the earth. During the current study, it has been found that the genus possess plenty of flavonoids, tannin and other polyphenolics which extends its biological efficacy towards antiinflammatory, antimicrobial, anticancerous and other therapeutic potentials. It was found in most of the reports and reviews that were surveyed in the present investigation, the crude extract (aqueous or organic fractions) to be potential agent for various activities. However, thorough examination needs to be carried out to see the efficacy and activity of individual component and in combination to explore the synergistic effects, if any.
